# Transduced PEP-1-Heme Oxygenase-1 Fusion Protein Attenuates Lung Injury in Septic Shock Rats

**DOI:** 10.1155/2018/6403861

**Published:** 2018-02-27

**Authors:** Xue-Tao Yan, Xiang-Hu He, Yan-Lin Wang, Zong-Ze Zhang, Jun-Jiao Tang

**Affiliations:** ^1^Department of Anesthesiology, Shenzhen Bao'an Maternity and Child Health Hospital, Shenzhen 518133, China; ^2^Department of Anesthesiology, Zhongnan Hospital, Wuhan University, Wuhan, Hubei 430071, China

## Abstract

Oxidative stress and inflammation have been identified to play a vital role in the pathogenesis of lung injury induced by septic shock. Heme oxygenase-1 (HO-1), an effective antioxidant and anti-inflammatory and antiapoptotic substance, has been used for the treatment of heart, lung, and liver diseases. Thus, we postulated that administration of exogenous HO-1 protein transduced by cell-penetrating peptide PEP-1 has a protective role against septic shock-induced lung injury. Septic shock produced by cecal ligation and puncture caused severe lung damage, manifested in the increase in the lung wet/dry ratio, oxidative stress, inflammation, and apoptosis. However, these changes were reversed by treatment with the PEP-1-HO-1 fusion protein, whereas lung injury in septic shock rats was alleviated. Furthermore, the septic shock upregulated the expression of Toll-like receptor 4 (TLR4) and transcription factor NF-*κ*B, accompanied by the increase of lung injury. Administration of PEP-1-HO-1 fusion protein reversed septic shock-induced lung injury by downregulating the expression of TLR4 and NF-*κ*B. Our study indicates that treatment with HO-1 protein transduced by PEP-1 confers protection against septic shock-induced lung injury by its antioxidant, anti-inflammatory, and antiapoptotic effects.

## 1. Introduction

Septic shock is a subset of sepsis in which particularly profound circulatory, cellular, and metabolic abnormalities are associated with a greater risk of mortality than in sepsis alone [1]. Septic shock-associated acute lung injury remains the major cause of mortality in critically ill patients. Although the pathophysiology of septic shock is not entirely understood, it is known that an immune response to an infection plays a key role in the development of septic shock [2]. In addition, oxidative stress, inflammatory mediators, mitochondrial damage, and other mechanisms are also thought to be involved in this process [2, 3].

Heme oxygenase-1 (HO-1) is a rate-limiting enzyme in the heme degradation pathway and in the maintenance of iron homeostasis. HO-1 catabolizes the prooxidant heme molecule to form equimolar quantities of carbon monoxide (CO), biliverdin (BV; which is rapidly converted into bilirubin (BR)), and free iron (which leads to the induction of ferritin, an iron-binding protein) [4]. Accumulating evidence suggests that HO-1 confers protection against a variety of oxidant-induced cell and tissue injuries. Administration of HO-1 by pharmacological induction or gene transfer provided protection in a variety of preclinical models [4–6]. Moreover, protective effects have been demonstrated in HO-1 knockout mice and a human case of genetic HO-1 deficiency [7, 8]. Beneficial effects of HO-1 are not only mediated via enzymatic degradation of proinflammatory free heme but also via the downstream products of heme degradation, CO, BV, and BR [9]. Administration of exogenous CO, BV, or BR may substitute for the cytoprotective effects of HO-1 to confer protection in a variety of clinically applicable models [4, 10–12]. In addition, a variety of signaling molecules has been implicated in the cytoprotection conferred by HO-1, including the transcription factor nuclear factor-kappaB (NF-*κ*B), autophagic proteins, p38 mitogen-activated protein kinase, phosphatidylinositol 3-kinase/Akt, and others [5].

Toll-like receptors (TLRs) are components of the innate immune system that respond to exogenous and endogenous molecules. Interactions of TLRs with their ligands lead to the activation of downstream signaling pathways that induce an immune response by producing inflammatory cytokines, type I interferons (IFN), and other inflammatory mediators [13]. Ten TLR genes have been identified in humans (TLR1–TLR10) and 12 (TLR1–TLR9 and TLR11–TLR13) in mice [14]. Among them, TLR4 has been recognized as a key receptor on which both infectious and noninfectious stimuli converge to induce a proinflammatory response [15]. Some studies suggest that TLR4 is involved in acute hepatic injury induced by endotoxemia [16] and acute myocardial dysfunction caused by septic shock [17]. TLR4 signals act through two downstream pathways, ultimately leading to the activation of transcription factor NF-*κ*B. NF-*κ*B plays a critical role in triggering and coordinating both innate and adaptive immune responses, being a pivotal regulator of the inducible expression of key proinflammatory mediators [18]. The wide variety of genes regulated by NF-*κ*B includes those encoding cytokines, chemokines, adhesion molecules, acute phase proteins, and inducible effector enzymes [19]. Accumulating evidence supports the role of the TLR4/NF-*κ*B pathway in lung injury caused by various factors, including ventilators [20, 21], lipopolysaccharides (LPS) [22], and intestinal ischemia/reperfusion [23].

PEP-1 is one of the cell-penetrating peptides that efficiently improve the intracellular delivery of proteins into various cells and tissues [24]. In our previous study, we fused HO-1 protein with PEP-1 to obtain a PEP-1-HO-1 fusion protein, which was found to provide protective effects in intestinal or myocardial ischemia/reperfusion injury [25, 26]. However, it is not clear whether PEP-1-HO-1 fusion protein may protect against infectious disease-induced lung injury. Therefore, in the present study, we chose a model of septic shock in rats and investigated whether treatment with the PEP-1-HO-1 fusion protein protects against septic shock-induced lung injury.

## 2. Materials and Methods

### 2.1. Production of PEP-1-HO-1 Fusion Protein

Gene fragments of PEP-1 and HO-1 were synthesized and cloned into a vector plasmid (pET-15b) to construct the recombinant plasmid pET-15b-PEP-1-hHO-1. PEP-1-HO-1 fusion protein was produced and purified as previously described [25].

### 2.2. Animals and Groups

Thirty-two male, specific pathogen-free Sprague-Dawley rats weighing 210–260 g (6–8 weeks old) were purchased from the Department of Laboratory Animal Center of Wuhan University and kept under standardized conditions for food, water, light, and temperature. Rats were allowed to drink water but were fasted for 12 h before the study. This study conformed to the Guide for the Care and Use of Laboratory Animals of the National Institutes of Health, and the experimental procedures were approved by the Institutional Animal Ethics Committee of Zhongnan Hospital of Wuhan University. Rats were randomly divided into four groups (*n* = 8): sham group, cecal ligation and puncture group (CLP group), CLP + low-dose PEP-1-HO-1 fusion protein group (LD group), and CLP + high-dose PEP-1-HO-1 fusion protein group (HD group).

### 2.3. Surgical Procedures

All animals were anesthetized by intraperitoneal injection of 1% sodium pentobarbital (50 mg/kg). A polyethylene catheter was inserted into the right carotid artery for monitoring the mean arterial pressure. A cannula in the left iliac vein was used for the administration of the PEP-1-HO-1 fusion protein or physiological saline. Using sterile technique, the rats underwent a midline laparotomy. The cecum was carefully exteriorized and ligated below the ileocecal valve using 4–0 silk ligatures. The cecum was punctured twice by an 18-gauge needle before fecal contents leaked into the peritoneum. Then, the cecum was placed back into the abdominal cavity, and the incision was closed. In the LD group, rats were given PEP-1-HO-1 fusion protein (0.3 mg) via the left iliac vein at 1 h before CLP and 5 h after CLP, whereas rats in the HD group were given PEP-1-HO-1 fusion protein (0.6 mg) in the same way. Dose and time of the given PEP-1-HO-1 fusion protein were determined according to our previous results [25]. Instead of the PEP-1-HO-1 fusion protein, the equal volume of physiological saline was provided in the sham and CLP groups. In the sham group, the cecum was not ligated or punctured after laparotomy. All rats were subjected to the subcutaneous injection of physiological saline (2 mL/100 g) after operation. Twelve hours after CLP, the animals were sacrificed with pentobarbital overdose, blood samples were collected via the right carotid artery for subsequent measurement of serum HO-1 and cytokines, and tissue samples from the lung were harvested for further analysis.

### 2.4. Determination of Hemodynamic Parameters

At 0, 4, 8, and 12 h after CLP or sham surgery, the carotid artery catheter was connected to a pressure transducer (Smiths Medical ASD Inc., OH, USA), and mean arterial blood pressure (MAP) and heart rate (HR) were displayed on a polygraph recorder.

### 2.5. Histopathological Assessment

For evaluating the effects of the administration of the PEP-1-HO-1 fusion protein on histological changes of the lung, the harvested left upper lung lobe was immersed in 10% formalin for at least 24 h. After processing the lung tissues with standard methods, the samples were stained with hematoxylin and eosin. Two investigators observed these samples in a double-blind fashion. The histological lung injury was scored by the degrees of tissue edema, hemorrhage, and interstitial inflammatory cell infiltration [27]: 0 = no evidence, 1 = mild injury, 2 = moderate injury, and 3 = severe injury.

### 2.6. Determination of the Wet/Dry Weight Ratio

The left lower lung lobe was harvested and weighted at once after the rats were sacrificed. The sample was subsequently placed into a drying oven at 65°C for 48 h for the determination of the dry weight. The level of lung tissue edema was measured by calculating the wet/dry (W/D) weight ratio.

### 2.7. Determination of Lung Tissue Myeloperoxidase Activity

For assessing the polymorphonuclear neutrophil accumulation, the myeloperoxidase (MPO) activity was tested. Lung tissue samples were homogenized on ice using a homogenizer. The MPO activity was measured quantitatively according to the manufacturer's instructions (Jiancheng Biologic Project Company, Nanjing, China). One MPO activity unit was defined as the amount of lung tissue that converted 1 *μ*mol hydrogen peroxide to water per minute at 37°C. The MPO activity was expressed as units per gram (U/g) wet weight.

### 2.8. Determination of Lung Tissue Malondialdehyde Level and Superoxide Dismutase Activity

Frozen lung samples were homogenized in physiological saline and centrifuged at 4000*g* for 10 min at 4°C. Subsequently, the supernatant was received and assayed for its malondialdehyde (MDA) level and superoxide dismutase (SOD) activity. Spectrophotometrical measurements were performed according to the manufacturer's instructions by using commercial kits (Jiancheng Bioengineering Institute, Nanjing, China). The MDA level was expressed as nmol/mg protein, and the SOD activity was expressed as U/mg protein.

### 2.9. Determination of Serum HO-1 and Cytokines

The levels of HO-1, tumor necrosis factor alpha (TNF-*α*), and interleukin 6 (IL-6) in serum were examined using ELISA techniques. After collection and centrifugation of the blood samples, supernatants were collected for further analysis. The levels of serum HO-1, TNF-*α*, and IL-6 were quantified using ELISA kits (R&D, Minneapolis, MN, USA) according to the manufacturer's instructions. The concentrations of HO-1, TNF-*α*, and IL-6 were expressed as pg/mL.

### 2.10. Western Blot Analysis

The experiment was terminated by performing Western blot analysis of collected lung tissue samples, as described in the literature [25]. The samples were homogenized, lysed, and centrifuged using differential centrifugation for obtaining different protein components. Protein concentrations were measured by a BCA protein assay kit (Beyotime Institute of Biotechnology, Shanghai, China). Proteins were subjected to 10% sodium dodecyl sulfate-polyacrylamide gel electrophoresis and transferred to polyvinylidene fluoride membranes (Millipore, Bedford, MA, USA). Membranes were incubated with the primary antibodies against His-probe, cleaved caspase-3, Bcl-2, Bax, NF-*κ*Bp65, phosphorylated NF-*κ*Bp65 (p-NF-*κ*Bp65), and TLR4 (Santa Cruz Biotechnology, CA, USA) overnight at 4°C. Then, the membranes were washed in phosphate-buffered saline (0.05%). Tween 20 and proteins bands were visualized with an enhanced chemiluminescence kit (Amersham, Piscataway, NJ, USA). All band densities were quantified by densitometry using the Quantity One software (BioRad, Hercules, CA, USA).

### 2.11. Survival Study

In the mortality studies, some of the animals in each group (*n* = 12 per group) were assigned to a subgroup for survival analyses. The survival rates of the rats were recorded every 12 h for three days, as mentioned above, to observe whether the PEP-1-HO-1 fusion protein treatment confers protection against septic shock-induced lung injury.

### 2.12. Statistical Analysis

Statistical analysis was performed with the SPSS software version 22, and all data were expressed as mean ± SEM. The significance of differences in the measured values between groups was analyzed using an independent sample *t*-test, one-way analysis of variance (ANOVA), or two-way ANOVA for repeated measures followed by the Student–Newman–Keuls test. Histological scores were compared using the Kruskal–Wallis test. Survival analyses were performed using the Kaplan-Meier test followed by a log–rank test. *P* < 0.05 was considered statistically significant difference.

## 3. Results

### 3.1. Transduction of PEP-1-HO-1 Fusion Protein in Serum and Lung Tissues

**Figure 1:**
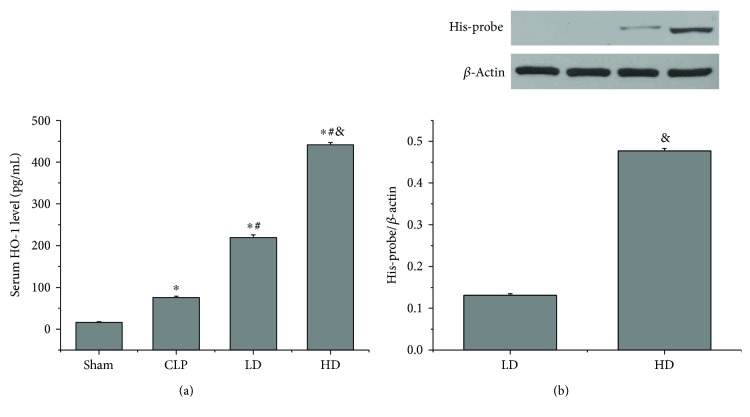
Analysis of the transduction of intravenous administration of PEP-1-HO-1 fusion protein. (a) Serum HO-1 level (*n* = 8 rats per group). (b) Expression of His-probe protein in lung tissues (*n* = 6 rats per group). Data are expressed as mean ± SEM. ^∗^*P* < 0.05 versus the sham group, ^#^*P* < 0.05 versus the CLP group, and ^&^*P* < 0.05 versus the LD group.

To determine the effective transduction of PEP-1-HO-1 fusion protein after intravenous administration of PEP-1-HO-1, we analyzed the serum HO-1 level by the ELISA and His-probe expression in lung tissues via Western blot analysis. CLP caused the increase of the serum HO-1 level (*P* < 0.05). Compared with the CLP group, the serum HO-1 levels in the LD and HD groups were significantly higher (*P* < 0.05). The level of serum HO-1 was higher in the HD group than in the LD group (*P* < 0.05; Figure 1(a)). As the His-probe protein is a part of the PEP-1-HO-1 fusion protein, its expression may be employed as an indicator for the expression of transduced HO-1 protein. Western blot analysis showed that the expression of His-probe protein was only found in PEP-1-HO-1-transduced animals and that this expression was dose-dependent, whereas no His-probe protein was detected in other groups (*P* < 0.05; Figure 1(b)). Our results indicated that PEP-1-HO-1 fusion protein was successfully delivered into the lungs.

### 3.2. Effects of PEP-1-HO-1 Fusion Protein on Mean Arterial Pressure and Heart Rate

The baseline values for MAP and HR were comparable in all groups. CLP led to a statistically significant, substantial attenuation of MAP at 12 h (*P* < 0.05), whereas significant and sustained increases in HR were observed from 4 h onwards after CLP (*P* < 0.05). The PEP-1-HO-1 treatment significantly prevented severe hypotension at 12 h after CLP and tachycardia from 4 h onwards after CLP (*P* < 0.05). Rats in the sham group exhibited stable hemodynamic conditions during the experimental period (*P* > 0.05; Tables 1 and 2).

### 3.3. Effects of PEP-1-HO-1 Fusion Protein on Histological Injury

**Figure 2:**
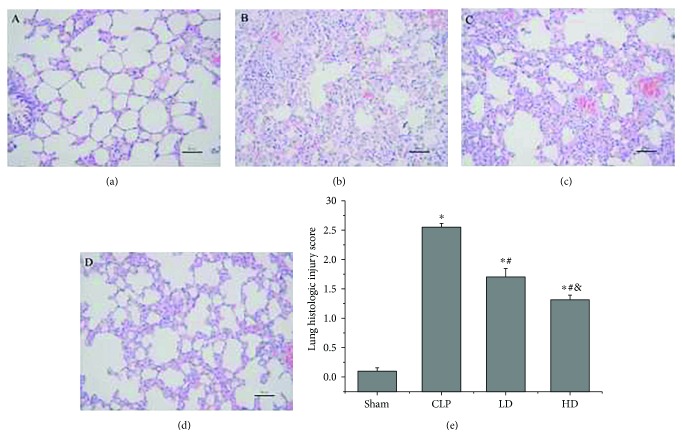
Histological changes in lung tissues stained with hematoxylin and eosin and histological injury scores of lung tissues in all groups. (a) Sham group. (b) Cecal ligation and puncture group (CLP group). (c) CLP + low-dose PEP-1-HO-1 fusion protein group (LD group). (d) CLP + high-dose PEP-1-HO-1 fusion protein group (HD group; original magnification ×200; scale bar = 50 *μ*m). (e) Histological injury score of the lung. Data are expressed as mean ± SEM; *n* = 8 rats per group. ^∗^*P* < 0.05 versus the sham group, ^#^*P* < 0.05 versus the CLP group, and ^&^*P* < 0.05 versus the LD group.

To assess the histological changes induced by the treatment protocols, histological injury was independently scored by two pathologists, and the average score was used for analysis (Figure 2). Normal histological structure was seen in the lung of the sham group (Figure 2(a)). The animals in the CLP group exhibited significant histological injury, including tissue edema, hemorrhage, and interstitial inflammatory cell infiltration (Figure 2(b)). In contrast, histological injury was alleviated in the animals subjected to the PEP-1-HO-1 fusion protein in the LD (Figure 2(c)) and HD groups (Figure 2(d)). Compared with the sham group, the injury scores of the lung tissues in the CLP, LD, and HD groups were significantly higher (*P* < 0.05). Compared with the CLP group, the injury scores of lung tissues in the LD and HD groups were significantly lower (*P* < 0.05). The injury score was lower in the HD group than in the LD group (*P* < 0.05; Figure 2(e)).

### 3.4. Effect of PEP-1-HO-1 Fusion Protein on the W/D Weight Ratio

**Figure 3:**
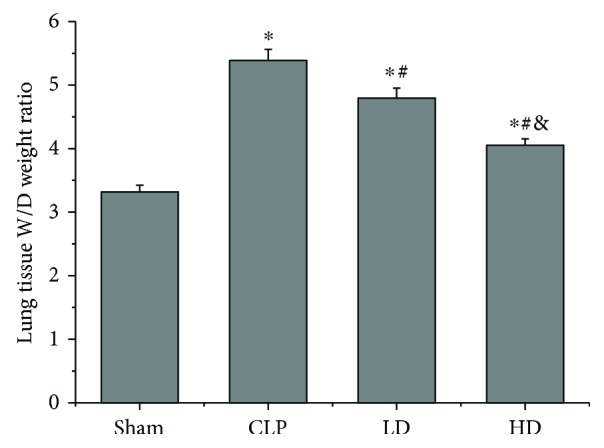
Effects of PEP-1-HO-1 fusion protein on the wet/dry (W/D) weight ratio in lung tissues. Data are expressed as mean ± SEM; *n* = 8 rats per group. ^∗^*P* < 0.05 versus the sham group, ^#^*P* < 0.05 versus the CLP group, and ^&^*P* < 0.05 versus the LD group. CLP: cecal ligation and puncture; LD: CLP + low-dose PEP-1-HO-1 fusion protein; HD: CLP + high-dose PEP-1-HO-1 fusion protein.

The lung tissue W/D weight ratio was higher in the three groups that underwent CLP than in the sham group (*P* < 0.05). Compared with the CLP group, the W/D weight ratio was remarkably lower in the LD and HD groups (*P* < 0.05) and lower in the HD group than in the LD group (*P* < 0.05; Figure 3).

### 3.5. Effects of PEP-1-HO-1 Fusion Protein on MPO Activity

**Figure 4:**
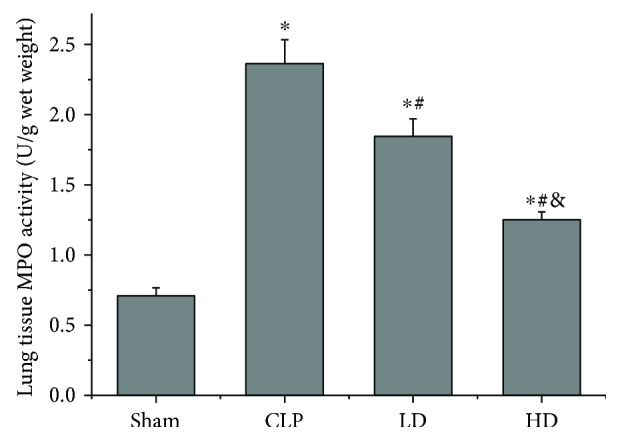
Effects of PEP-1-HO-1 fusion protein on myeloperoxidase (MPO) activity in lung tissues. Data are expressed as mean ± SEM; *n* = 8 rats per group. ^∗^*P* < 0.05 versus the sham group, ^#^*P* < 0.05 versus the CLP group, and ^&^*P* < 0.05 versus the LD group. CLP: cecal ligation and puncture; LD: CLP + low-dose PEP-1-HO-1 fusion protein; HD: CLP + high-dose PEP-1-HO-1 fusion protein.

The activity of MPO, an enzyme present in neutrophils, was used as a marker of neutrophil infiltration. The lung tissue MPO activity was higher in the three groups that underwent CLP than in the sham group (*P* < 0.05). In contrast, administration of the PEP-1-HO-1 fusion protein halted this augmentation (*P* < 0.05), and the MPO activity was lower in the HD group than in the LD group (*P* < 0.05; Figure 4).

### 3.6. Effects of PEP-1-HO-1 Fusion Protein on MDA Level and SOD Activity

**Figure 5:**
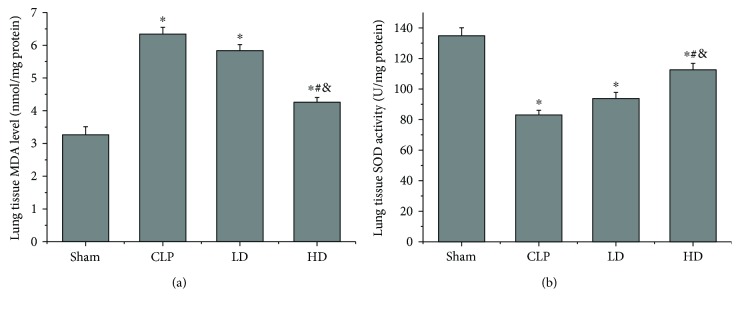
Effects of PEP-1-HO-1 fusion protein on malondialdehyde (MDA) level and superoxide dismutase (SOD) activity in lung tissues. (a) MDA level. (b) SOD activity. Data are expressed as mean ± SEM; *n* = 8 rats per group. ^∗^*P* < 0.05 versus the sham group, ^#^*P* < 0.05 versus the CLP group, ^&^*P* < 0.05 versus the LD group. CLP: cecal ligation and puncture; LD: CLP + low-dose PEP-1-HO-1 fusion protein; HD: CLP + high-dose PEP-1-HO-1 fusion protein.

CLP significantly increased the tissue MDA level (*P* < 0.05) and decreased the SOD activity (*P* < 0.05). Compared with the CLP group, treatment with high-dose PEP-1-HO-1 fusion protein significantly reduced the increased MDA level (*P* < 0.05) and enhanced the decreased SOD activity (*P* < 0.05). However, treatment with low-dose PEP-1-HO-1 fusion protein had no influence on MDA level and SOD activity (*P* > 0.05). Compared with the LD group, treatment with high-dose PEP-1-HO-1 fusion protein significantly reduced the increased MDA level (*P* < 0.05) and enhanced the decreased SOD activity (*P* < 0.05; Figure 5).

### 3.7. Effects of PEP-1-HO-1 Fusion Protein on Serum TNF-*α* and IL-6 Levels

**Figure 6:**
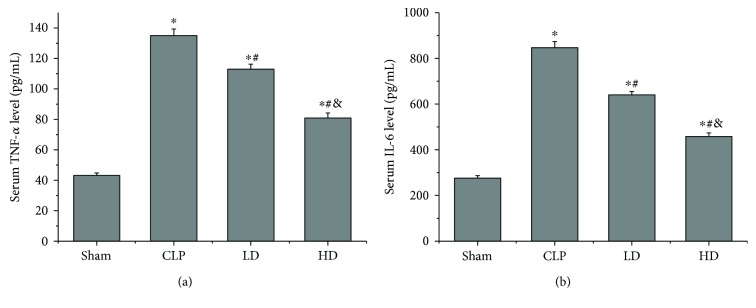
Effects of PEP-1-HO-1 fusion protein on serum tumor necrosis factor alpha (TNF-*α*) and interleukin 6 (IL-6) levels in all groups. (a) Serum TNF-*α* level. (b) Serum IL-6 level. Data are expressed as mean ± SEM; *n* = 8 rats per group. ^∗^*P* < 0.05 versus the sham group, ^#^*P* < 0.05 versus the CLP group, and ^&^*P* < 0.05 versus the LD group. CLP: cecal ligation and puncture; LD: CLP + low-dose PEP-1-HO-1 fusion protein; HD: CLP + high-dose PEP-1-HO-1 fusion protein.

Blood samples were harvested to measure the concentrations of TNF-*α* and IL-6 in serum. The levels of serum TNF-*α* and IL-6 in rats subjected to CLP were higher than those in the sham group (*P* < 0.05). Compared with the CLP group, serum TNF-*α* and IL-6 levels in the LD and HD groups were significantly reduced (*P* < 0.05), and the serum TNF-*α* and IL-6 levels were lower in the HD group than in the LD group (*P* < 0.05; Figure 6).

### 3.8. Effects of PEP-1-HO-1 Fusion Protein on the Expressions of Cleaved Caspase-3, Bcl-2, and Bax

**Figure 7:**
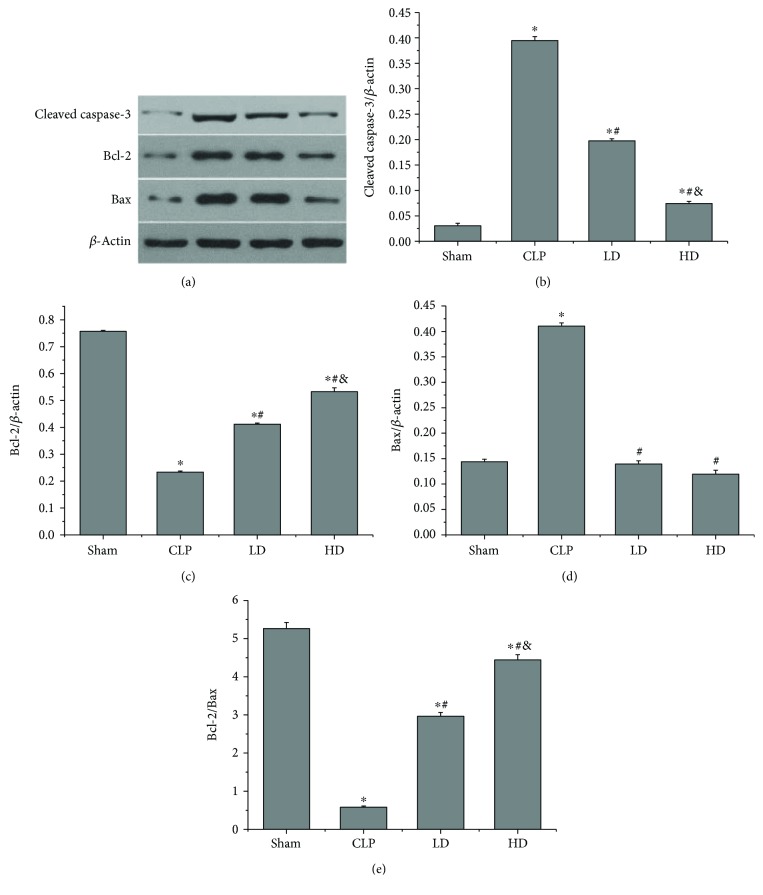
Effects of PEP-1-HO-1 fusion protein on the expression of cleaved caspase-3, Bax, and Bcl-2 in all groups. (a) The expressions of cleaved caspase-3. Bcl-2 and Bax in lung tissues were detected by Western blot analysis. (b) Cleaved caspase-3 expression. (c) Bcl-2 expression. (d) Bax expression. (e) Bcl-2/Bax ratio. Data are expressed as mean ± SEM; *n* = 6 rats per group. ^∗^*P* < 0.05 versus the sham group, ^#^*P* < 0.05 versus the CLP group, and ^&^*P* < 0.05 versus the LD group. CLP: cecal ligation and puncture; LD: CLP + low-dose PEP-1-HO-1 fusion protein; HD: CLP + high-dose PEP-1-HO-1 fusion protein.

To determine the possible role of cell apoptosis in lung injury induced by CLP, we tested the expression of proapoptotic and antiapoptotic proteins in lung tissues by Western blot analysis. CLP markedly upregulated the expressions of cleaved caspase-3 and Bax but reduced the Bcl-2 expression (*P* < 0.05). The Bcl-2/Bax ratio was significantly decreased in septic shock rats (*P* < 0.05). Compared with the CLP group, the expressions of cleaved caspase-3 and Bax were significantly downregulated, the Bcl-2 expression was increased, and the Bcl-2/Bax ratio was enhanced in the LD and HD groups (*P* < 0.05). Compared with the LD group, the expressions of cleaved caspase-3 and Bax were significantly reduced, the Bcl-2 expression was increased, and the Bcl-2/Bax ratio was enhanced in the HD group (*P* < 0.05; Figure 7).

### 3.9. Effects of PEP-1-HO-1 Fusion Protein on NF-*κ*B and TLR4 Expression

**Figure 8:**
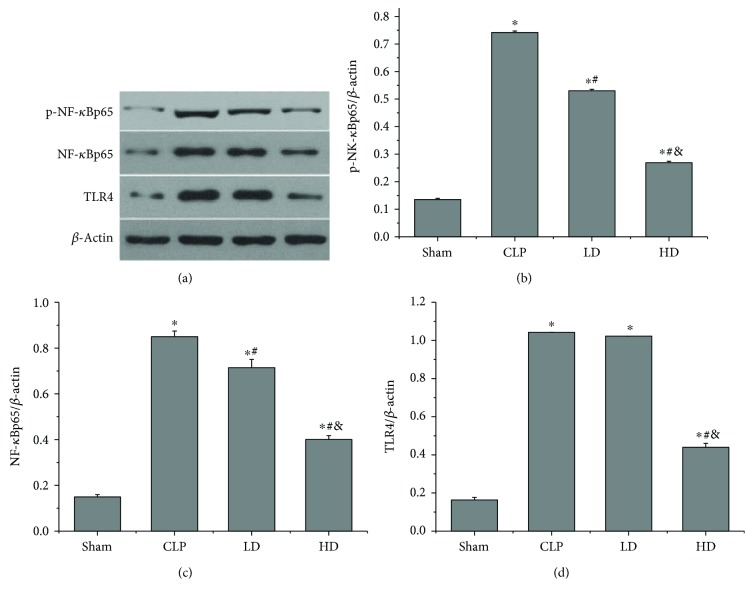
Effects of PEP-1-HO-1 fusion protein on the expression of nuclear factor-kappaBp65 (NF-*κ*Bp65), phosphorylated NF-*κ*Bp65 (p-NF-*κ*Bp65), and Toll-like receptor 4 (TLR4) in lung tissues. (a) The expressions of NF-*κ*Bp65, p-NF-*κ*Bp65, and TLR4 in lung tissues were detected by Western blot analysis. (b) p-NF-*κ*Bp65 expression. (c) NF-*κ*Bp65 expression. (d) TLR4 expression. Data are expressed as mean ± SEM; *n* = 6 rats per group. ^∗^*P* < 0.05 versus the sham group, ^#^*P* < 0.05 versus the CLP group, and ^&^*P* < 0.05 versus the LD group. CLP: cecal ligation and puncture; LD: CLP + low-dose PEP-1-HO-1 fusion protein; HD: CLP + high-dose PEP-1-HO-1 fusion protein.

For exploring the possible protective mechanism of the PEP-1-HO-1 fusion protein against lung injury in septic shock rats, we tested the expressions of NF-*κ*Bp65 and TLR4 in lung tissues by Western blot analysis and detected remarkable differences among all groups. CLP markedly upregulated the expressions of NF-*κ*Bp65, p-NF-*κ*Bp65, and TLR4 (*P* < 0.05). Compared with the CLP group, the expressions of NF-*κ*Bp65 and p-NF-*κ*Bp65 were significantly decreased in the LD and HD groups (*P* < 0.05); the expression of TLR4 was reduced in the HD group (*P* < 0.05), but not in the LD group (*P* > 0.05). The values of NF-*κ*Bp65, p-NF-*κ*Bp65, and TLR4 in the HD group were lower than those in the LD group (*P* < 0.05; Figure 8).

### 3.10. Effect of PEP-1-HO-1 Fusion Protein on Survival Rate

**Figure 9:**
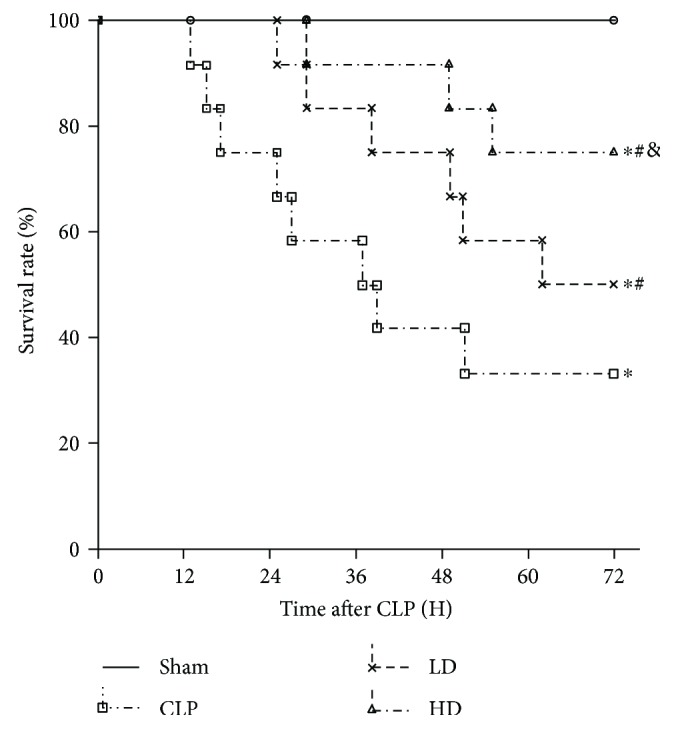
Effects of PEP-1-HO-1 fusion protein on the survival rate in septic shock rats. Kaplan-Meier survival curves of rats that received sham operation (sham group), cecal ligation and puncture plus saline (CLP group), CLP plus low-dose PEP-1-HO-1 fusion protein (LD group), and CLP plus high-dose PEP-1-HO-1 fusion protein (HD group). Data are expressed as the percentage of rats that survived at the observed time point. *n* = 12 rats per group. ^∗^*P* < 0.05 versus the sham group, ^#^*P* < 0.05 versus the CLP group, and ^&^*P* < 0.05 versus the LD group.

To determine if the PEP-1-HO-1 fusion protein influences the survival rate, we analyzed the mortality at designated time points. No mortality was observed within 72 h in all sham animals. The survival rate was significantly decreased to 33.33% at 72 h after CLP. In contrast, the rats in the LD and HD groups had higher survival rates of 50% and 75% at 72 h after CLP. These results suggested that PEP-1-HO-1 treatment significantly improved the 72 h survival rate of CLP rats (*P* < 0.05; Figure 9).

## 4. Discussion

In this study, we have demonstrated in a rat model that CLP-induced septic shock caused significant lung injury, as evidenced by pathologic morphological changes seen in the lung tissues as well as the increased the lung wet/dry ratio. We found that treatment with the PEP-1-HO-1 fusion protein dose-dependently reduced lung morphological damage and alleviated injuries induced by oxidative stress, inflammatory reactions, and apoptosis. Furthermore, we found that administration of PEP-1-HO-1 fusion protein inhibited the increased expressions of TLR4 and NF-*κ*Bp65 in septic shock rats. Our data indicated that the PEP-1-HO-1 fusion protein conferred a protection against lung injury induced by septic shock in rats.

The reasons for choosing HO-1 as a therapeutic agent were its antioxidant, anti-inflammatory, and antiapoptotic properties and its important protective role in multiple organ damage, such as heart [28], lung [5], and gastrointestinal injury [29]. In addition, some studies indicate that protective effects of many pharmacological agents may be in part mediated by HO-1 upregulation [30–34]. Thus, in the present study, we used protein transduction technology to transduce the HO-1 protein into septic shock animals for investigating the protective effects of HO-1 on lung injury. Because we chose a 12 h, CLP-induced septic shock as our experimental model, the therapeutic agent, PEP-1-HO-1 fusion protein was given twice (once every 6 h). Dose and time of the administration of the PEP-1-HO-1 fusion protein were chosen according to our previous study [25]. We found that treatment with the PEP-1-HO-1 fusion protein obviously attenuated the septic shock-induced lung injury and that its protective effects were dose-dependent.

Although the exact mechanisms of septic shock-induced lung injury have not been fully elucidated yet, it is universally accepted that oxidative stress plays a vital role in this process [35]. MDA, a product of lipid peroxidation, is usually considered an index of the degree of oxidative damage. SOD is the primary enzymatic method to reduce superoxides. MDA and SOD are frequently used to evaluate cell injury. In this study, we found that septic shock caused the increase in the MDA level and the decrease in the SOD activity, thus exacerbating lung injury. It is well documented that many antioxidants have been used for the treatment of sepsis-induced acute lung injury in animal models [36, 37] and in patients with sepsis [38]. Our results showed that transduced exogenous HO-1 protein significantly reduced the increase of the MDA level and suppressed the decrease of the SOD activity, thus alleviating septic shock-induced lung injury. Therefore, our data indicate that oxidative stress caused by septic shock may be a crucial part of lung injury, and HO-1 may attenuate septic shock-induced oxidative stress to contribute to lung protection.

Apoptosis is a critical process that causes timely death in senescent cells, which is important in developmental biology and in the pathophysiology of various diseases. In this study, we found that septic shock led to the increase of the cleaved caspase-3 expression and the decrease of the Bcl-2/Bax ratio, accompanied by the increase of the lung injury score and the lung wet/dry ratio. In contrast, treatment with the PEP-1-HO-1 fusion protein reversed these changes and consequently prevented lung injury in septic shock animals. A study found that apoptotic adipose-derived mesenchymal stem cell therapy reduced inflammatory, oxidative, and apoptotic (Bax, caspase-3) biomarkers, increased antiapoptotic (Bcl-2) and antioxidant biomarkers, and protected against lung and kidney injury in the sepsis syndrome caused by CLP in rats [39]. Perl et al. [40] found that hemorrhagic shock and sepsis increased the expression of caspase-3 and altered the lung histology. Contrarily, administration of caspase-3 siRNA not only attenuated lung apoptosis but also ameliorated acute lung injury. In addition, it is well known that apoptosis may amplify inflammatory responses [41]. Our study found that administration of PEP-1-HO-1 fusion protein alleviated apoptosis-mediated lung injury, accompanied by the decrease of the MPO activity in lung tissues. It is well accepted that MPO activity is used as a marker of neutrophil infiltration. Furthermore, infiltrated neutrophils may directly cause apoptosis. Tsao et al. [42] found that administration of levosimendan had beneficial effects on multiple organ injury caused by peritonitis-induced septic shock by decreasing neutrophil infiltration and attenuating caspase-3 expression. Ahmad et al. [43] also found that treatment with NaHS alleviated CLP-induced septic shock and protected against multiple organ failure by attenuating CLP-induced increases in the MPO and plasma levels of proinflammatory mediators. Our experimental results are consistent with this conclusion.

In addition, proinflammatory cytokines released by damaged tissues are thought to play a vital role in the pathogenesis of septic shock-induced lung injury. In this study, we investigated the levels of TNF-*α* and IL-6 in the serum and found that these levels were higher in the CLP group than in the sham group. Treatment with PEP-1-HO-1 fusion protein significantly decreased the increased TNF-*α* and IL-6 levels. It is not sure whether elevated levels of cytokines derived from the gut or lungs caused lung damage, because we detected increased levels of TNF-*α* and IL-6 in the lungs and intestine (data not shown). However, a study found that inflammatory cytokines from damaged intestines were responsible for the damage of remote organs [44]. Furthermore, we also found that septic shock caused the increase of the p-NF-*κ*Bp65 expression in the lungs. NF-*κ*B is a crucial transcription factor, which regulates the expression of various genes related to inflammation and immune response, including many proinflammatory factors such as TNF-*α*, IL-1, and IL-6 [2, 45]. Weng et al. [46] found that honokiol, a low molecular weight natural product possessing anti-inflammatory activity, rescued sepsis-associated acute lung injury by reversing NF-*κ*B activation and decreasing the production of serum TNF-*α*. In the present study, we found that administration of PEP-1-HO-1 fusion protein inhibited NF-*κ*B activation in lung tissues and significantly decreased the levels of TNF-*α* and IL-6 in serum, thus alleviated lung injury. Our results were in accordance with previous studies, indicating that treatment with PEP-1-HO-1 fusion protein reduced septic shock-induced lung injury via inhibition of the production of proinflammatory cytokines regulated by activated NF-*κ*B.

TLRs are a family of pattern recognition receptors, playing a pivotal role in the cellular activation of the innate immune response. TLRs have been identified to be involved in lung injury induced by various factors, including I/R, hemorrhagic, or septic shock [22, 23]. In this study, we found that septic shock obviously resulted in a significant increase of the TLR4 expression in lung tissues, accompanied by the increase of the lung injury score and the lung wet/dry ratio. Our results were consistent with previous reports. In addition, various studies have demonstrated that some therapeutic strategies have been shown to protect against lung injury by inhibiting the activation of the TLR4/NF-*κ*B pathway [47, 48]. Sun et al. [22] demonstrated that sevoflurane, a new halogen-containing volatile anesthetic, directly exerted anti-inflammatory effects on lung injury caused by LPS in vitro and in vivo via inhibition of the TLR4/NF-*κ*B pathway. In the present study, we found that treatment with the PEP-1-HO-1 fusion protein notably reduced the septic shock-induced increase of TLR4 expression and NF-*κ*B activation, which attenuated inflammation, oxidative stress, and apoptosis and ameliorated lung injury. These results were in accordance with previous studies indicating that protective effects of the PEP-1-HO-1 fusion protein on septic shock-induced lung injury might be partially due to inhibition of the TLR4/NF-*κ*B pathway activation.

The present study has some limitations. First, we mainly investigated the protective effects of the PEP-1-HO-1 fusion protein on septic shock-induced lung injury in rats. However, cell-penetrating peptides have been shown to induce dose- and length-dependent cytotoxic effects. Thus, it is necessary to assess the potential hazard of the cell-penetrating peptide PEP-1 itself to those tissues and cells that need to be protected in our future study. Secondly, although we found that treatment with the PEP-1-HO-1 fusion protein significantly reduced the septic shock-induced increase of the TLR4 and NF-*κ*B expressions, there was insufficient evidence to draw the conclusion that the PEP-1-HO-1 fusion protein provides a protective role against septic shock-induced lung injury through suppressing the activation of the TLR4/NF-*κ*B pathway. Further study using antagonists/agonists or deficient animals is needed.

## 5. Conclusion

This study provides evidence that transduced PEP-1-HO-1 fusion protein may reduce the septic shock-induced increase of TLR4 expression and NF-*κ*B activation and alleviate septic shock-induced lung injury through the antioxidant, anti-inflammatory, and antiapoptotic properties of transduced HO-1. Our study provides a new option for the treatment of main organ damage caused by infectious diseases.

## Figures and Tables

**Table 1:** Changes in mean arterial pressure at specific time points for each group (mmHg, *n* = 8).

Group	0 h	4 h	8 h	12 h
Sham	127.5 ± 2.02	121.00 ± 2.54	119.25 ± 3.32	116.63 ± 3.72
CLP	126.63 ± 1.94	111.75 ± 2.04	92.75 ± 3.23^∗^	74.25 ± 4.35^∗^
LD	122.25 ± 3.12	113.63 ± 2.24	101.13 ± 3.14^∗^	89.13 ± 3.24^∗^^,#^
HD	124.88 ± 2.47	117.88 ± 2.83	106.25 ± 3.23^∗^^,#^	97.5 ± 1.96^∗^^,#^

Values are expressed as mean ± SEM. ^∗^*P* < 0.05 versus the sham group; ^#^*P* < 0.05 versus the CLP group. CLP: cecal ligation and puncture; LD: CLP + low-dose PEP-1-HO-1 fusion protein; HD: CLP + high-dose PEP-1-HO-1 fusion protein.

**Table 2:** Changes in heart rate at specific time points for each group (beats/min, *n* = 8).

Group	0 h	4 h	8 h	12 h
Sham	327.13 ± 4.38	328.00 ± 4.02	324.13 ± 4.31	325.25 ± 3.30
CLP	326.88 ± 3.82	369.50 ± 5.10^∗^	388.25 ± 3.09^∗^	395.38 ± 4.28^∗^
LD	324.63 ± 4.15	352.88 ± 3.92^∗^^,#^	367.50 ± 3.22^∗^^,#^	376.75 ± 3.74^∗^^,#^
HD	331.75 ± 3.43	348.88 ± 2.89^∗^^,#^	362.75 ± 3.72^∗^^,#^	369.50 ± 3.17^∗^^,#^

Values are expressed as mean ± SEM. ^∗^*P* < 0.05 versus the sham group; ^#^*P* < 0.05 versus the CLP group. CLP: cecal ligation and puncture; LD: CLP + low-dose PEP-1-HO-1 fusion protein; HD: CLP + high-dose PEP-1-HO-1 fusion protein.
